# Factors associated with health-related quality of life in renal transplant recipients: results of a national survey in France

**DOI:** 10.1186/1477-7525-11-88

**Published:** 2013-05-30

**Authors:** Stéphanie Gentile, Davy Beauger, Elodie Speyer, Elisabeth Jouve, Bertrand Dussol, Christian Jacquelinet, Serge Briançon

**Affiliations:** 1 Laboratoire de Santé Publique, Faculté de Médecine, Université Aix-Marseille, Marseille EA 3279, France; 2 Service Santé Publique et Information Médicale, CHU Marseille, Marseille, France; 3 Centre de Néphrologie et de Transplantation Rénale, CHU Marseille, Marseille, France; 4 Agence de la Biomédecine, Paris, France; 5 Epidémiologie et Evaluation Cliniques, CHU Nancy, Nancy, France; 6 Université de Lorraine, Université Paris Descartes, Apemac, Nancy EA 4360, France

**Keywords:** Associated factors, Cross-sectional multicenter study, Quality of life, Renal transplant recipient, ReTransQol, SF-36

## Abstract

**Background:**

This study aims to identify factors associated with health related quality of life (HRQOL) through a comprehensive analysis of sociodemographic and clinical variables among a representative sample size of renal transplant recipients (RTR) in France.

**Methods:**

A cross-sectional multicenter study was carried out in 2008. All RTR over 18 years old with a functioning graft for at least one year were included. Data included socio-demographic, health status, and treatment characteristics. To evaluate HRQOL, the Short Form-36 Health Survey (SF-36) and a HRQOL instrument for RTR (ReTransQol) were administered. Multivariate linear regression models were performed.

**Results:**

A total of 1061 RTR were included, with a return rate of 72.5%. The variance explained in regression models of SF-36 ranges from 20% to 40% and from 9% to 33% for ReTransQol.

The variables which decreased scores of both HRQOL questionnaires were: females, unemployment, lower education, living alone, high BMI, diabetes, recent critical illness and hospitalization, non-compliance, a long duration of dialysis and treatment side effects.

Specific variables which decreased ReTransQol scores were dismissal and a recent surgery on the graft. These which decreased SF36 scores were being old and a recent infectious disease.

The variables the most predictors of worse HRQOL were: side effects, infectious disease, recent hospitalization and female gender.

**Conclusions:**

The originality of our study’s findings was that novel variables, particularly treatment side effects and unemployment, have a negative effect on quality of life of RTR. The French Biomedicine Agency and the National Health Institute for Public Health Surveillance conduct specific actions for professional reintegration and therapeutic education programs in the national plan to improve the HRQOL of people living with chronic diseases.

## Introduction

In public health and in medicine, the concept of health-related quality of life (HRQOL) is defined as an individual’s perception of their position in life in the context of the culture and value systems in which they live and in relation to their goals, expectations, standards and concerns [1]. Physicians have often used HRQOL to measure the effects of chronic illness in their patients to better understand how an illness interferes with a person’s day-to-day life [2,3].

Although the HRQOL advantages of renal transplantation are well established [4-8], large differences of quality of life are often observed depending on specific transplant cohorts [9,10]. Life after renal transplantation presents negative aspects as well, such as a strict regimen of immunosuppressive drugs and its related side effects, frequent medical visits, infections, the uncertainty and anxiety concerning rejection episodes and potential loss of the graft [11-16]. Therefore, one of the most important issues for the future of transplantation is to more clearly specify the full range of personal, environmental and clinical factors that negatively influence HRQOL outcomes. A better understanding of the role of these factors is essential to develop interventions that maximize the HRQOL in the context of transplantation. The World Health Organization (WHO) prioritizes HRQOL improvement for people living with chronic diseases, End-stage renal disease patients were concerned by a specific WHO program [17]. In France, the August 9, 2004 public health law applied this priority, implementing a national plan to improve the HRQOL for people living with chronic diseases [18]. The French Biomedicine Agency and the National Institute for Public Health Surveillance have promoted studies to determine the level of HRQOL of end-stage renal disease patients in France, at the start of the national plan to improve the HRQOL of chronic disease patients [19,20].

In France, dialysis patients are followed-up through the French Renal Epidemiology and Information Network (REIN) [21]. REIN began in 2002 to provide a tool for public health decision support, evaluation and research related to renal replacement therapies for end-stage renal disease. It relies on a network of nephrologists, epidemiologists, patients and public health representatives coordinated regionally and nationally. Moreover, the renal transplant recipients (RTR) are registered in a transplant database (CRISTAL) managed by the French Biomedicine Agency [22], which collects social and medical data for all patients who receive an organ transplant. Both databases are updated regularly, and have been used as sources of data for the end-stage renal disease specific program to improve HRQOL.

Two separate studies planned as part of the “QUAVI-REIN” (the French translation for Renal Quality of Life) multiregional projects have been performed in 8 of the 22 regions of France: one focusing on dialysis patients [23], and the second focusing on RTR when data were available for both populations. This study aims to identify factors associated with HRQOL through a comprehensive analysis of sociodemographic and clinical variables among a representative sample size of RTR in France.

## Materials and methods

### Data sources

A cross-sectional multicenter study was carried out in France between March 2007 and March 2008, in the eight regions of France participating in the REIN network in 2003: Auvergne, Bretagne, Champagne-Ardennes, Languedoc-Roussillon, Limousin, Lorraine, Provence-Alpes-Côte d’Azur and Rhône-Alpes.

### Participants

All RTR over 18 year of age with a functioning graft for at least one year were eligible. Multi-organ transplant patients before or simultaneously with their kidney transplant were excluded. RTR and their addresses were identified from the CRISTAL database. The sample was stratified by regions and age class, using the same sampling rate for each stratum. The sample size was calculated to detect a difference of 5 points in the Short Form-36 Health Survey (SF-36) HRQOL score considering a standard deviation of 20, assuming a two-sided level of 5% and 80% power. A maximum of four comparisons was scheduled. The sample size calculation was 1,000 patients. Considering a non-response rate of approximately 30%, 1,300 questionnaires were sent in order to achieve 1,000 patients. We randomized an additional sample of 500 patients in case the response rate in some region or age class was not adequate. A total of 401 patients were not included: 155 were returned to sender, 3 deceased patients and 243 were lost to follow-up. Finally, 162 more questionnaires were sent to complete a lack of data in some age class, reaching a total of 1462 questionnaires sent in all 8 regions. The number of self-administrated questionnaires returned was 1061.

#### Measures

Data collection included demographic, socio-demographic, medical characteristics and HRQOL. All data were obtained directly from patients except their age, gender and nephropathy, which were obtained from the CRISTAL database.

### Socio-demographic characteristics

Demographic variables assessed were gender, age, employment status (retirement, unemployment and working), education level (primary or less, secondary 1st stage and higher than secondary), living arrangement (living alone versus other status) and dismissal due to illness (when an employer did a procedure for dismissing a member of staff due to illness).

### Medical characteristics

Medical measures were grouped into three domains related to kidney disease, health status, and treatment (i.e. drugs, side effects and compliance).

1. Kidney disease: etiology of end-stage renal failure, duration of dialysis, duration of transplantation and graft rejection episodes.

2. Health status: comorbidities (i.e. hypertension, Diabetes Mellitus), intercurrent health events during the last four weeks, critical illness, especially infectious disease, graft surgery, hospital admission, Body Mass Index (BMI), and smoking status.

3. Treatment: to collect the drug treatments, we established with nephrologists a selective list of the most commonly prescribed drugs using their generic names.

The patient could also add the medication if drugs were not included in the list. Therapeutics data were classified according to Anatomical Therapeutic and Chemical (ATC) classification [[24]].

4. Side effects: to collect treatment side effects, we used a list of 18 items identified by RTR [25]. The patient rated the importance on a 4-level Likert scale, ranging from “no discomfort” to “very significant discomfort”.

Side effects were categorized into 5 domains according to the following classification:

1. General health: muscular weaknesses, general tiredness, pain, lower limb oedema, etc.

2. Mental Health: depression, anxiety, sleep disorders.

3. Body modification: facial changes, facial oedema, weight gain, hair loss, swollen gums, brittle skin, etc.

4. Sexual dysfunctional

5. Diarrhea

Three items explored compliance:

• Difficulty to respect the immunosuppressant schedule

• Modification of immunosuppressive therapy

• Modification of the dosage of treatments (other than immunosuppressive therapy drugs)

Patients who answered “yes” to one of these three items were categorized as non-compliant.

### Health-related quality of life

SF-36 [26] and ReTransQol [27] were used to evaluate HRQOL.

The French version of the SF-36 is a generic instrument, with scores ranging from 0 (complete dissatisfaction) to 100 (full satisfaction) for eight domains: physical functioning (PF), role-physical (RP), bodily pain (BP), general health (GH), vitality (VT), social functioning (SF), role-emotional (RE) and mental health (MH). The correlated physical (PCS) and mental (MCS) summary components were computed following the standardized procedure provided by authors.

The ReTransQol is a specific instrument consisting of 45 items describing 5 dimensions: physical health (PH), mental health (MH), medical care and satisfaction (MC), treatment (TRT) and fear of losing graft (FG). Each score ranges from 0 to 100, and the higher the score, the better the perceived state of health [27].

### Data collection procedures

All measurement instruments were sent to the patient’s residence with a letter signed by the project coordinator. Patients returned the completed questionnaires via a pre-stamped envelope. Non-respondents were reminded by a second letter three weeks later and contacted by phone.

#### Ethical aspects

The study methodology was approved by the local Institutional Review Board (CCTIRS n°06-311) and the “Comité National Informatique et Liberté” (CNIL n°906248), which ensures the confidentiality of all information collected.

#### Statistical analysis

Continuous variables were expressed as mean ± standard deviation (SD) or range. Discrete variables were reported as frequency and percentage. Group comparisons were performed using analysis of variance (bivariate analysis). All factors with a p-value <0.25 were included as candidate variables in a multivariate analyses, according to the literature review.

Multivariate linear regression models (MLR) were used to estimate the relationship between HRQOL scores and socio-demographic, health status and treatment characteristics.

Multivariate analysis was summarized by the β coefficients and their 95% confidence interval and p-value. The R-squares were performed. The level of significance was set at a p-value <0.05 (factors presented). Statistical analysis was performed using SAS® 9.2 system software.

## Results

At the time of the survey, there were 5,991 patients present in the CRISTAL database living with a functional graft for at least one year in the 8 regions. Data referring to a total of 1061 patients were obtained from 1462 sampled, with a response rate of 72.5%.

### Socio-demographic characteristics

The mean age of patients was 55.2 years (± 12.4 years) and 61.8% were male. Nearly 80% of RTR lived as a couple. Less than 40% of patients were employed at the time of the survey. Among unemployed RTR, half were retired. Patient characteristics are shown in Table 1.

**Table 1:** Socio-demographic characteristics

	**n**	**%**
Male	656	61.8%
Age, years (mean ± SD)	55.2 ± 12.4	
**Level of education**		
Primary or less	260	25.8%
Secondary 1st stage (college & high school)	483	48%
Higher than secondary, 2nd stage or university	264	26.2%
**Living arrangement**		
Live alone	186	19.2%
**Employment status**		
Employed	377	35.5%
Unemployed	566	33.1%
Retired	118	31.4%

### Medical characteristics

Kidney disease: The mean time since transplantation was 8.5 years (± 5.8 years, range 1–40). A total of 23.5% of patients had at least one intercurrent health event in the past four weeks (Table 2). Among this sample, 11.1% (n = 111) had been hospitalized in the last four weeks, 4.7% (n = 47) had a critical illness, 13.7% (n = 123) had an infectious disease, 4% (n = 42) had acute rejection episodes and 1.9% (n = 17) had a surgery on the graft in the last four weeks.

**Table 2:** Medical characteristics and treatments (compliance and side effects)

		**n**	**%**
**Medical characteristics**	Cadaveric donor transplantation	1035	97.5%
	Duration of transplantation, years	8.5 ± 5.8	
	Patients with rejection since renal transplant	222	22.9%
	**Major causes of ESRD**		
	Chronic glomerular nephritis	380	35.8%
	Interstitial nephropathy	122	11.5%
	Hereditary nephropathy	206	19.4%
	Duration of dialysis, months (mean ± SD)	31.3 ± 37.2	
	**Intercurrent health events in the last four weeks**	**245**	**23.2%**
	Hospitalization	111	11.1%
	Critical illness	47	4.7%
	Infectious disease	123	13.7%
	Acute rejection episodes	42	4.0%
	Graft surgery	17	1.9%
	**Comorbidities**		
	Hypertension	844	80.9%
	BMI > 30 (kg/m^2^)	140	13.4%
	Diabetes mellitus	133	12.9%
	Current smokers	122	11.8%
**Treatments, compliance and side**	Side effects	838	79%
	Mean number of side effects per patient	5.7 ±3.9	
	Range	(1–17)	
	Mean number of drugs per patient	5.1 ±3.1	
	**Immunosuppressive treatment**		
	Calcineurin inhibitors	956	90.6%
	Corticosteroids	593	56.2%
	Antimetabolites	769	72.9%
	Proliferation inhibitors	53	5.0%
	Monotherapy	109	10.3%
	Bitherapy	564	53.5%
	Tritherapy	378	35.8%
	**Other treatment**		
	Antihypertensive drugs	890	84.4%
	Hypolipidemics	540	51.2%
	Antidiabetic agents	100	9.5%
	Non-compliant to treatment	239	22.5%
	**Side effects related to:**		
	General health	548	51.6%
	Mental health	467	44.0%
	Body modification	771	72.7%
	Sexual disorders	288	27.1%
	Diarrhea	216	20.4%

### Treatment

Most patients (89.3%) had two or three immunosuppressive drugs. Nearly 84% of patients had antihypertensives, 51.2% had lipid lowering drugs and 9.5% antidiabetic agents. Most of the patients (77.5%) were compliant. Difficulty to respect the medication regimen was the main reason for non-compliance. The majority of patients (79%) reported treatment side effects, particularly those related to body modification: 72.7% (n = 771). The mean number of side effects was 5.7 ± 3.9 (range 1–17) (Table 2).

### Health related quality of life

Table 3 shows mean HRQOL scores and SD corresponding to the different dimensions of the SF-36 and ReTransQol questionnaires used.

**Table 3:** HRQOL scores

**Dimensions**	**Means ± SD**
**S F - 3 6**	
Physical Functioning- PF	74.8 ± 24.3
Social Functioning- SF	74.9 ± 23.6
Role Physical- RP	64.4 ± 41.3
Role Emotional- RE	68.8 ± 41.3
Mental Health	65.5 ± 18.7
Vitality- VT	53.3 ± 19.3
Bodily Pain- BP	68.3 ± 25.8
General Heath- GH	55.4 ± 21
Physical Component Summary- PCS	45.8 ± 9.7
Mental Component Summary- MCS	46.0 ± 10.5
**R e T r a n s Q o l**	
Physical Health - PH	63.8 ± 17.4
Mental Health - MH	72.6 ± 16.7
Medical Care - MC	75.0 ± 14.9
Fear of losing the Graft - FG	58.4 ± 20.4
Treatment - TRT	70.7 ± 13.9

#### Variables included the final regression model for both HRQOL questionnaires

In SF-36 and ReTransQol models of regression, at least 16 and 14 variables, respectively, were included to strengthen the model and obtain significant scores, with a good variance explanation for each dimension. Adjusted differences in the eight generic scales of SF-36 and in the five specific dimensions of ReTransQol using socio-demographic, medical and treatment variables are shown in Tables 4 and 5. The variance explained in regression models for SF-36 ranges from 20% to 40% (Table 4) and from 9% to 33% for ReTransQol (Table 5).

**Table 4:** Final regression models (SF 36 domains)

**Dimensions**	**Variables**	**β coeff.**	**95% CI**	**P values**
**PF** R^2^ = 0.31	Intercept	95.4	[91.1; 99.6]	p < .0001
	Female	-4.7	[-7.6; -1.7]	p < 0.0018
	Age ≥ 75 years	-23.3	[-30.4; -16.2]	p < .0001
	Low educational (primary or less)	-7.5	[-11.2; -3.8]	p < .0001
	Unemployment	-6.3	[-9.4; -3.2]	p < .0001
	BMI > 30 (kg/m^2^)	-5.8	[-10; -1.6]	p < 0.0065
	Critical illness in the last 4 weeks	-8.0	[-14.9; -1.1]	p < 0.0237
	Diabetes	-5.6	[-10.1; -1]	p < 0.0163
	Side effects related to general health	-17.0	[-20.9; -13.1]	p < .0001
	Side effects related to mental health	-6.8	[-10.5; -3]	p < 0.0004
	Recent surgery	-9.5	[-24.9; 6]	p < 0.2283
	Infectious disease in the last 4 weeks	-4.8	[-9.1; -0.4]	p < 0.0311
**RP** R^2^ = 0.22	Intercept	90.5	[82.8; 98.1]	p < .0001
	Female	-6.9	[-12.2; -1.6]	p < 0.0104
	Age ≥ 75 years	-20.1	[-33.1; -7]	p < 0.0026
	Low educational (primary or less)	-11.4	[-18; -4.8]	p < 0.0007
	Hospitalization in the last 4 weeks	-17.9	[-27.1; -8.8]	p < 0.0001
	Critical illness in the last 4 weeks	-31.5	[-44.8; -18.1]	p < .0001
	Infectious disease in the last 4 weeks	-10.9	[-19; -2.9]	p < 0.0080
	Side effects related to general health	-21.5	[-28.7; -14.3]	p < .0001
	Side effects related to mental health	-16.0	[-22.8; -9.1]	p < .0001
**BP** R^2^ = 0.23	Intercept	85.3	[80.6; 90]	p < .0001
	Female	-4.9	[-8.1; -1.6]	p < 0.0035
	Age ≥ 75 years	-8.3	[-16.1; -0.4]	p < 0.0402
	Low educational (primary or less)	-6.5	[-10.5; -2.5]	p < 0.0016
	Infectious disease in the last 4 weeks	-9.1	[-14; -4.3]	p < 0.0002
	Side effects related to general health	-18.7	[-23.2; -14.2]	p < .0001
	Side effects related to mental health	-9.7	[-14; -5.4]	p < .0001
	Side effects related to body modification	-4.2	[-7.7; -0.7]	p < 0.0193
**MH** R^2^ = 0.24	Intercept	81.1	[77.7; 84.6]	p < .0001
	Female	-5.6	[-8; -3.3]	p < .0001
	Family status (living alone)	-4.6	[-7.2; -1.9]	p < 0.0007
	Duration of dialysis > 3 years	-3.3	[-5.8; -0.8]	p < 0.0087
	Hospitalization in the last 4 weeks	-7.7	[-11.4; -4]	p < .0001
	Side effects related to mental health	-16.5	[-19.5; -13.5]	p < .0001
	Side effects related to body modification	-4.4	[-6.9; -1.9]	p < 0.0005
**RE** R^2^ = 0.18	Intercept	94.8	[86.5; 103]	p < .0001
	Female	-6.4	[-12; -0.8]	p < 0.0252
	Low educational (primary or less)	-16.8	[-24; -9.6]	p < .0001
	Age ≥ 75 years	-11.1	[-25.3; 3.2]	p < 0.1285
	Family status (living alone)	-9.1	[-15.3; -2.8]	p < 0.0045
	Hospitalization in the last 4 weeks	-17.4	[-26.7; -8.1]	p < 0.0003
	Infectious disease in the last 4 weeks	-19.1	[-27.6; -10.7]	p < .0001
	Side effects related to mental health	-25.0	[-32; -18.1]	p < .0001
**SF** R^2^ = 0.25	Intercept	90.8	[86.8; 94.8]	p < .0001
	Female	-4.9	[-7.6; -2.1]	p < 0.0006
	Family status (living alone)	-4.6	[-7.8; -1.5]	p < 0.0036
	Hospitalization in the last 4 weeks	-11.8	[-16.2; -7.3]	p < .0001
	Infectious disease in the last 4 weeks	-7.2	[-11.5; -2.9]	p < 0.0011
	Diabetes	-7.5	[-11.5; -3.5]	p < 0.0002
	Side effects related to general health	-11.3	[-15; -7.6]	p < .0001
	Side effects related to mental health	-16.4	[-20; -12.8]	p < .0001
**VT** R^2^ = 0.22	Intercept	62.8	[59.6; 66.1]	p < .0001
	Female	-2.4	[-4.6; -0.1]	p < 0.0366
	Hospitalization in the last 4 weeks	-8.3	[-11.8; -4.8]	p < .0001
	Side effects related to general health	-12	[-15; -8.9]	p < .0001
	Side effects related to mental health	-11.7	[-14.8; -8.7]	p < .0001
	Side effects related to body modification	-3.9	[-6.3; -1.4]	p < 0.0020
**GH** R^2^ = 0.18	Intercept	63.8	[62.1; 65.5]	p < .0001
	Hospitalization in the last 4 weeks	-6.1	[-10.2; -2]	p < 0.0038
	Critical illness in the last 4 weeks	-7.1	[-13.5; -0.7]	p < 0.0305
	Diabetes	-5.4	[-9; -1.7]	p < 0.0038
	Side effects related to general health	-11.6	[-15.1; -8.1]	p < .0001
	Side effects related to mental health	-7.7	[-11.1; -4.2]	p < .0001
	Side effects related to body modification	-5.6	[-8.4; -2.9]	p < .0001
	Non-compliant	-6.3	[-9.2; -3.4]	p < .0001
**PCS** (R^2^ = 0.28)	Intercept	52.7	[50.9; 54.5]	p < .0001
	Female	-1.7	[-3; -0.4]	p < 0.0084
	Age ≥ 75 years	-7.7	[-11; -4.3]	p < .0001
	Low educational (primary or less)	-3.3	[-4.9; -1.7]	p < .0001
	Unemployment	-2.5	[-3.8; -1.1]	p < 0.0003
	Critical illness in the last 4 weeks	-5.7	[-8.8; -2.6]	p < 0.0003
	Infectious disease in the last 4 weeks	-3.2	[-5.1; -1.3]	p < 0.0012
	Side effects related to general health	-9.2	[-10.8; -7.5]	p < .0001
**MCS** (R^2^ = 0.22)	Intercept	52.1	[50.3; 54]	p < .0001
	Female	-1.7	[-3; -0.4]	p < 0.0113
	Family status (living alone)	-2.2	[-3.7; -0.7]	p < 0.0039
	Hospitalization in the last 4 weeks	-5.7	[-7.8; -3.6]	p < .0001
	Side effects related to body modification	-2.5	[-3.9; -1.1]	p < 0.0005
	Side effects related to mental health	-9.3	[-11; -7.7]	p < .0001

**Table 5:** Final regression models (ReTransQol domains)

**Dimensions**	**Variables**	**B coeff.**	**95% CI**	**P values**
**PH** R^2^ = 0.31	Intercept	78.2	[75.2; 81.2]	p < .0001
	Female	-2.5	[-4.5; -0.5]	p < 0.0156
	Low educational (primary or less)	-7.3	[-9.6; -5.1]	p < .0001
	BMI > 30 (kg/m^2^)	-4.6	[-7.4; -1.7]	p < 0.0021
	Unemployment	-7.2	[-9.4; -5.1]	p < .0001
	Duration of dialysis > 3 years	-2.5	[-4.7; -0.4]	p < 0.0226
	Hospitalization in the last 4 weeks	-5.2	[-8.6; -1.8]	p < 0.0029
	Critical illness in the last 4 weeks	-6	[-10.6; -1.4]	p < 0.0110
	Side effects related to general health	-9.8	[-12.5; -7]	p < .0001
	Side effects related to mental health	-6	[-8.7; -3.3]	p < .0001
	Side effects related to body modification	-4.4	[-6.6; -2.2]	p < .0001
**MH** R^2^ = 0.27	Intercept	87	[84.1; 89.9]	p < .0001
	Female	-4.1	[-6.1; -2.2]	p < .0001
	Family status (living alone)	-8.1	[-10.3; -5.8]	p < .0001
	Dismissal	-3.7	[-6.7; -0.7]	p < 0.0152
	Duration of dialysis > 3 years	-3.9	[-5.9; -1.8]	p < 0.0003
	Hospitalization in the last 4 weeks	-5.1	[-8.3; -2]	p < 0.0014
	Recent surgery	-10.3	[-18.2; -2.4]	p < 0.0107
	Side effects related to general health	-6.4	[-9.1; -3.7]	p < .0001
	Side effects related to mental health	-10.2	[-12.8; -7.5]	p < .0001
	Side effects related to body modification	-3.7	[-5.9; -1.6]	p < 0.0007
**MC** R^2^ = 0.1	Intercept	79.2	[78; 80.5]	p < .0001
	Family status (living alone)	-3.8	[-5.9; -1.7]	p < 0.0004
	Dismissal	-3.1	[-6; -0.2]	p < 0.0354
	Side effects related to general health	-5.9	[-8.4; -3.3]	p < .0001
	Side effects related to mental health	-3.6	[-6.1; -1]	p < 0.0056
	Side effects related to body modification	-2.9	[-4.9; -0.8]	p < 0.0057
**TR** R^2^ = 0.31	Intercept	77.4	[76.4; 78.4]	p < .0001
	BMI > 30 (kg/m^2^)	-3.2	[-5.3; -1.1]	p < 0.0030
	Non-compliant	-4.4	[-6.1; -2.7]	p < .0001
	Side effects related to general health	-9.5	[-11.5; -7.5]	p < .0001
	Side effects related to mental health	-6.8	[-8.8; -4.8]	p < .0001
	Side effects related to body modification	-7.5	[-9.1; -5.9]	p < .0001
**FG** R^2^ = 0.1	Intercept	66.5	[62.3; 70.7]	p < .0001
	Female	-3	[-5.8; -0.1]	p < 0.0409
	Side effects related to mental health	-11.4	[-15.1; -7.7]	p < .0001
	Side effects related to body modification	-4.9	[-7.9; -1.8]	p < 0.0016

### Factors associated with a modification of HRQOL for SF-36

The variables which were associated with lower SF-36 scores were: older age, female gender, unemployment, lower education, living alone, high BMI, diabetes, infectious disease, critical illness and hospitalization in the last 4 weeks, non-compliance, former smoker, a long duration of dialysis, side effects related to general health and mental health or body modification domains (Table 4).

**Figure 1:**
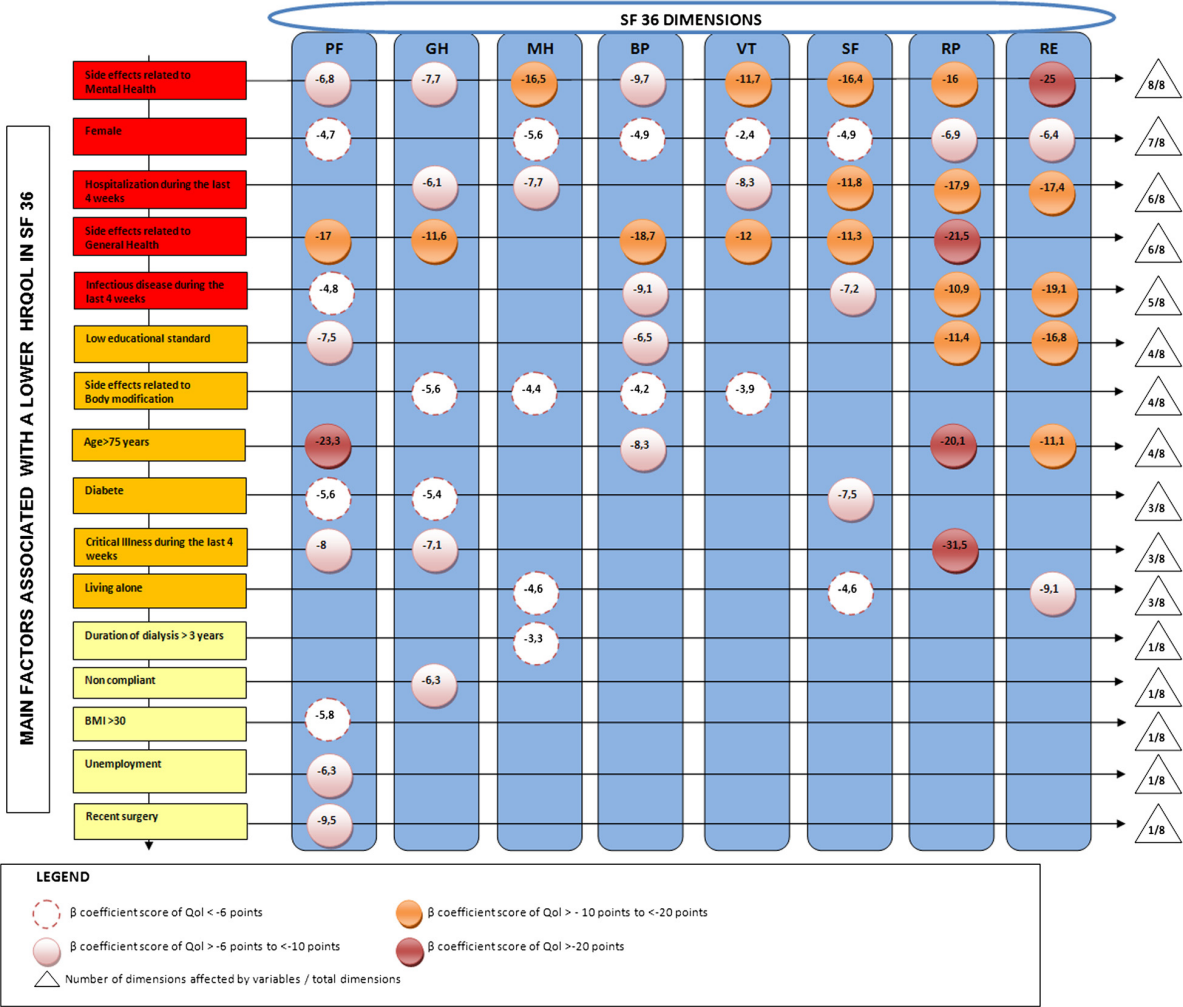
Matrix diagram for SF36.

The five variables which contributed most to a worse quality of life were: side effects related to general health and mental health domains, infectious disease, hospitalization in the last 4 weeks, and female gender (Figure 1). The “potentially modifiable” variables are in bold and the others are consider as “not likely modifiable” by intervention programs.

### Factors associated with a modification of HRQOL for retransQOL

**Figure 2:**
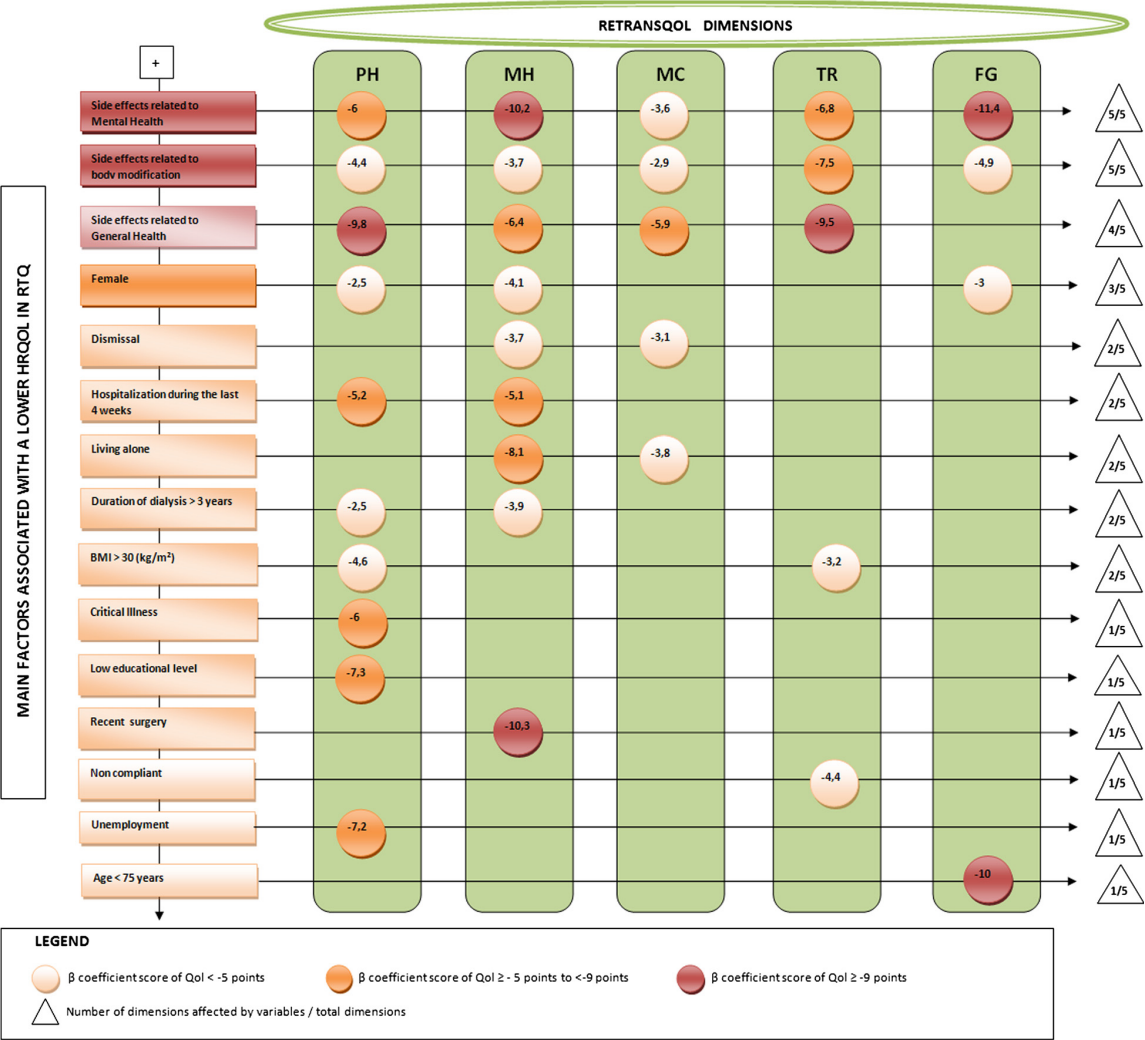
Matrix diagram for RTQ.

The following variables (Table 5) played a significant role in the reduction of HRQOL using the ReTransQol subscales: female gender, unemployment, dismissal, lower education, living alone, high BMI, side effects related to general health, mental health and body modification domains, infectious disease, critical illness, hospitalization in the last 4 weeks, non-compliant, a recent surgery on the graft and a long duration of dialysis (Figure 2).

The four variables which were associated with a lower quality of life are those associated to side effects related to mental health, body modifications and general health domains, and female gender.Discussion

We analyzed the factors associated with a lower quality of life in a representative sample of 1,061 renal transplant recipients from 8 regions of France. This study is the first report in France of HRQOL in kidney transplantation with such a large sample of patients.

The sociodemographic characteristics of our sample were similar to those reported in the literature [28-30]. Our sample is representative of general French RTR. Moreover, there are few studies with extensive observations carried out. In our literature review, few studies had a sample of over 1,000 patients [6,31,32]. Therefore, the substantial sample size, the response rate of 72.8%, and multivariate analysis are strengths of the present study.

After an analyze of the literature review, the level of quality of life of our patients may be different [4] as well as similar [33-36], depending of the country of the study, the sample size, the medical care or the Public Health organization. In general, SF36 scores are slightly lower than those found in the literature, probably because of our big sample size of patients used compared with other studies.

Another strong point of the current study was the construction of a comprehensive multivariate model, including several sociodemographic, clinical and treatment variables in its adjusted analysis. The independent variables were selected based on a previous univariate analysis and from a literature review. The final regression models explained 32% of the physical (PCS) HRQOL variance and 23% of mental HRQOL variance (MCS). Previous studies describe that use of highly effective predictive regression models only explained between 3% and 22% of HRQOL variance among chronic renal disease patients [37,38].

As in other studies, we used a generic questionnaire of HRQOL associated with a specific one. We applied the generic instrument SF-36 Health Survey, the most used questionnaire for HRQOL analysis in RTR [2,39,40] We also associated a disease-specific instrument validated for RTR in the French language: the ReTransQol [27].

In our study, quality of life scores were lower with all the socio-demographic and health status variables collected. The impact of socio-demographic variables is known for having a negative influence on HRQOL. These findings are in accordance with other studies: level of HRQOL significantly decreases with age [41], gender, living status and the educational level [34,42-44].

This study points out that unemployed patients have an extremely impaired HRQOL, especially for physical and mental dimensions, whatever the measurement instrument used [43,45]. This is why the French Biomedicine Agency and the National Health Institute for Public Health Surveillance made it a priority to conduct specific actions for professional reintegration, related to their plan to improve the quality of life of chronic disease patients.

In accordance with other studies, comorbidities like a BMI over 30, the presence of diabetes, the duration of dialysis and the smoking status reduced the quality of life in almost all dimensions, but intercurrent events of health also have a negative influence on the HRQOL in all dimensions [46-48]. Among intercurrents events, having been hospitalized in the last 4 weeks, having an infectious disease and a critical illness in the last 4 weeks, and having a recent surgery on the graft are associated with a lower HRQOL.

Our results also emphasized the negative impact of treatment [49,50]. We found that compliance and side effects were associated with very low scores [51], in every dimension and regardless of the measurement instrument used [52-54]. These results could be explained by the lack of information received concerning possible side effects, and the extreme importance of the treatment schedule [55]. Perhaps the duration of doctor’s visits could be longer or more frequent, with specific health education about treatment including how the drugs must be taken and adhered to, treatment benefits, and side effects. Then, medical staff could propose specific programs to patients to handle the difficulties due to specific treatment, using a medical booklet regarding their treatment [56]. Thus, the French Biomedicine Agency and the National Health Institute for Public Health Surveillance have recommended a program for specific health education of RTR [57].

Concerning ReTransQol specific dimensions, the physical aspects of HRQOL of RTR have been reported as impaired. Physical restrictions are mostly associated with the frequent occurrence of comorbidities and side effects of immunosuppressive therapy [58,59]. However, we can observe that the ReTransQol specifically showed that medical variables are responsible for worsening health, especially the 3 main factors which are exclusively treatment side effects. Our study pointed out that older age has a positive influence on the fear of losing the graft dimension (β coeff. = −10 points for age < 75 years). Indeed, elderly patients were less anxious about the fear of losing their graft.

We can consider that the ReTransQol is more sensitive than SF-36 for health status variables, but less exhaustive for socio-demographic factors. Finally, it could be very interesting to work with both, because these tools are complementary and offer different views on HRQOL for RTR [60]. Limitations of our research are related to the study design (cross-sectional), so we cannot truly interpret predictive factors. A cohort study or panel study would allow us to analyze risk factors and use correlations to determine absolute predictive factors. However, this study is currently repeated under the same conditions to compare scores and its evolution over time.

## Conclusion

The clinical relevance of this study lies in demonstrating that comorbidities frequently occur as side effects of immunosuppressive therapy, such as hypertension and diabetes, which are associated with physical aspects of the HRQOL after renal transplantation. Therefore, the occurrence of these side effects should be taken into account in the choice of the renal replacement therapy (dialysis or transplantation). In addition, it is possible that public policies directed toward vocational rehabilitation may lead to positive effects in mental HRQOL. Intervention to improve mental HRQOL on personal factors indirectly related to health may increase the probability of professional rehabilitation, with personal as well as socioeconomic advantages. A better understanding of the role of personal factors is essential in the development of psychosocial interventions to maximize HRQOL. Given that treatment side effects negatively impact every level of HRQOL, we recommend a better medical follow-up of side effects, considering specificities related to gender. Having a professional activity seems to be a major element in improving the HRQOL of RTR. These actions are integrated into the national HRQOL improvement plan in France. Repetition of this study is necessary to measure the impact of actions, and is now underway with a larger sample including more than 20 regions in France.

### Summary

This study aims to identify factors associated with health related quality of life (HRQOL) through a comprehensive analysis of sociodemographic and clinical variables among a representative sample size of renal transplant recipients in France.

### Consent

“Written informed consent was obtained from the patient for publication of this report and any accompanying images”.

## Competing interests

The authors declare that they have no competing interests.

## Authors’ contributions

SG conceived the study and its design, coordinated the data management, analyzed and interpreted the data, drafted the manuscript; DB performed some statistical analysis, analyzed and interpreted the data and drafted the manuscript; ES participated to the interpretation of data and revised the manuscript. EJ participated in the design of the study, collected the data and performed the statistical analysis BD participated in the design of the study, collected medical data and participated to the interpretation of data CJ et SB revised the manuscript critically for important intellectual content and have given final approval of the version to be published. All authors read and approved the final manuscript.
